# CD209 signaling pathway as a biomarker for cisplatin chemotherapy response in small cell lung cancer

**DOI:** 10.1016/j.gendis.2023.06.011

**Published:** 2023-07-16

**Authors:** Anqi Lin, Hong Yang, Jian Zhang, Peng Luo

**Affiliations:** Department of Oncology, Zhujiang Hospital, Southern Medical University, Guangzhou, Guangdong 510282, China; Department of Oncology, Zhujiang Hospital, Southern Medical University, Guangzhou, Guangdong 510282, China; The First Clinical Medical School, Southern Medical University, Guangzhou, Guangdong 510515, China; Department of Oncology, Zhujiang Hospital, Southern Medical University, Guangzhou, Guangdong 510282, China

One of the bottlenecks in the clinical treatment of small cell lung cancer (SCLC) is its susceptibility to multidrug resistance. While SCLC is sensitive to platinum-based chemotherapy at the initial stage of treatment, it can rapidly become drug-resistant, allowing tumor growth to accelerate. A significant decrease in drug sensitivity after recurrence and multidrug resistance can seriously interfere with the effectiveness of treatment, making sensitivity maintenance one of the urgent challenges to be solved. Therefore, discovering prognostic markers for cisplatin chemotherapy and understanding the mechanisms of cisplatin resistance have great practical significance for improving the outcomes of SCLC patients. CD209, also known as DC-SIGN, belongs to the C-type lectin superfamily and is mainly expressed in dendritic cells. In the past few years, growing evidence has shown that CD209 can also combine with Lewis antigens (which are highly expressed in cancer) and promote the process of T-cell presentation, thus initiating a series of immune cascades.^1^ Damaging this process, which thus helps tumors evade the immune response, may be one of the possible mechanisms of chemotherapy resistance. However, controversy has been raised about the role of the CD209 signaling pathway in different types of cancer. In lung cancer, increased expression of CD209 is associated with better survival, while in colorectal cancer, the opposite trend is observed, with CD209 promoting cancer progression.^1^^,^^2^ In this study, we explored the relationships between the activation of the CD209 signaling pathway with the efficacy of cisplatin and prognosis in SCLC patients.

To investigate the activation state of the CD209 signaling pathway, we first obtained the gene set of the CD209 signaling pathway from the Molecular Signatures Database (MSigDB) (Table S1). Single-sample gene set enrichment analysis (ssGSEA) was then performed on the RNA-seq data of 45 SCLC patients (the Local-SCLC cohort) who were treated with cisplatin-based chemotherapy at Zhujiang Hospital of Southern Medical University during the period 2017–2020. This study was approved by the Research Ethics Committee of Zhujiang Hospital of Southern Medical University, with all participants providing written informed consent. A validation cohort was obtained from a study by George J et al^3^ that provided detailed clinical information for 68 SCLC patients receiving platinum-based chemotherapy (George-SCLC cohort). The clinical information of patients from the two cohorts is shown in Table S2 and 3. According to the median ssGSEA scores of the CD209 signaling pathway, the patients were divided into the CD209-High group and the CD209-Low group, representing different activation states of the CD209 signaling pathway. In the Local-SCLC cohort, univariate Cox regression analysis showed that up-regulation of the CD209 signaling pathway was associated with longer overall survival (OS) (Fig. 1A). Multivariate Cox analysis revealed that activation of the CD209 signaling pathway was an independent predictor of prognosis. Similarly, a consistent trend was observed in the George-SCLC cohort (Fig. S1). These findings were validated by Kaplan–Meier survival analysis, revealing that up-regulation of the CD209 signaling pathway was associated with longer OS following chemotherapy (Fig. 1B; Fig. S2).

**Figure 1:**
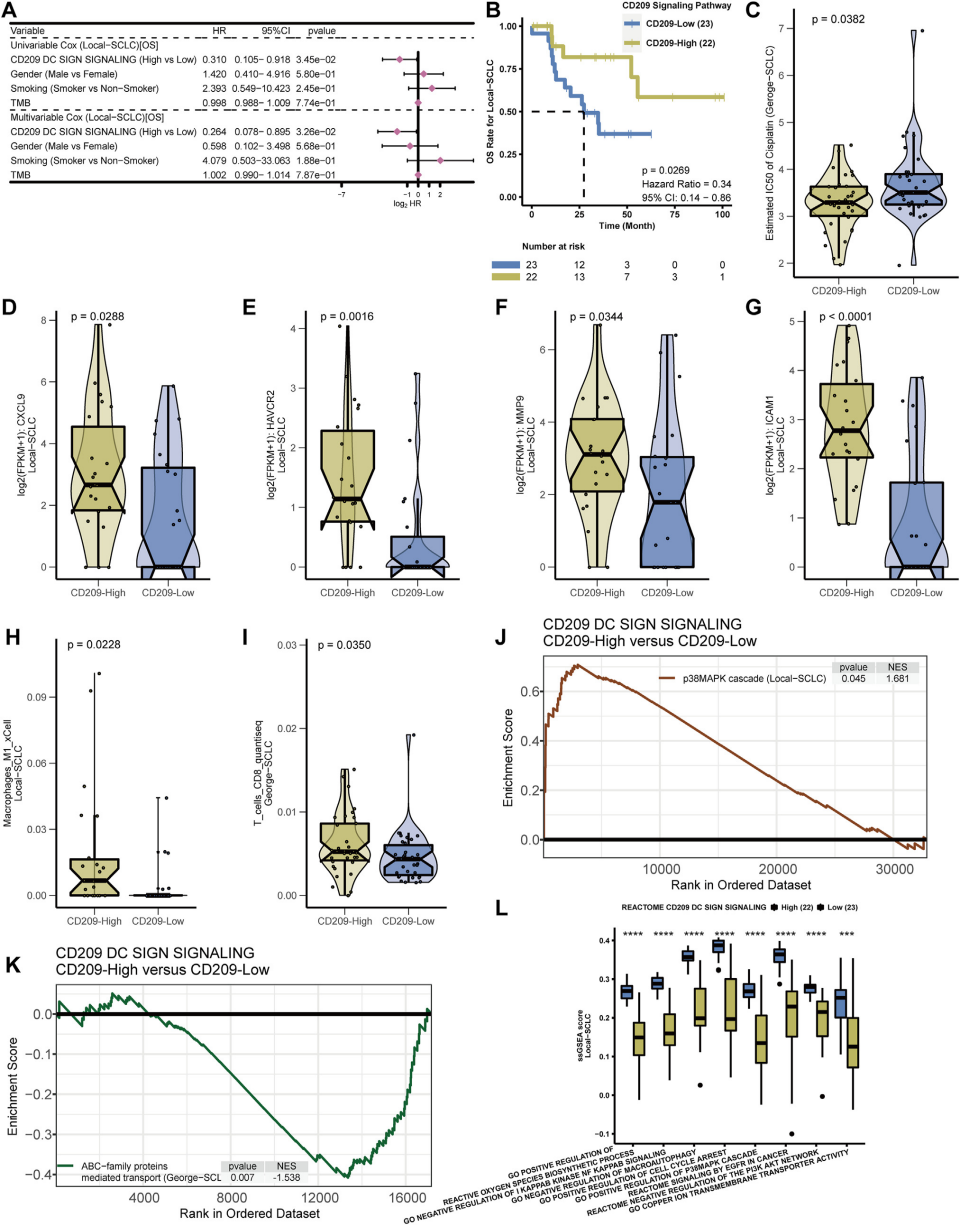
Value of the CD209 signaling pathway for predicting prognosis and cisplatin sensitivity in SCLC. **(A)** Univariate and multivariate Cox regression analyses showed that activation of the CD209 signaling pathway was associated with longer OS in the Local-SCLC cohort. **(B)** Kaplan–Meier survival analysis was performed to assess the relationship between the CD209 signaling pathway activation state and OS in patients in the Local-SCLC cohort. The patients were divided into two subgroups according to the median ssGSEA scores of the CD209 signaling pathway. The *p* value was calculated by the log-rank test. **(C)** Cisplatin sensitivity prediction analysis was performed for different CD209 signaling pathway activation states in patients from the George-SCLC cohort. **(D**–**G)** Comparison of immune-related molecules between groups with different CD209 signaling pathway activation states in Local-SCLC, in order of CXCL9, HAVCR2, MMP9, and ICAM1. **(H)** The violin plot shows the immune infiltration levels of M1 macrophages in the Local-SCLC cohort with different CD209 signaling pathway activation states. **(I)** The absolute fraction of CD8^+^ T cells, calculated by quanTIseq, was significantly higher in the CD209-High group. *P* values were calculated by the Wilcoxon rank-sum test. **(J)** GSEA plot shows the p38MAPK cascade pathway enrichment results in the Local-SCLC cohort. **(K)** GSEA plot shows the ABC family protein-mediated transport pathway enrichment results in the George-SCLC cohort. **(L)** ssGSEA enrichment results for multiple representative pathways from the Local-SCLC cohort are shown. The asterisks indicate differences in pathway enrichment between different CD209 signaling pathway activation states, which were detected using the Mann–Whitney *U* test. ∗∗∗∗*P* < 0.0001, ∗∗∗*P* < 0.001, ∗∗*P* < 0.01, ∗*P* < 0.05. CI, confidence interval; GSEA, gene set enrichment analysis; HR, hazard ratio; IC50, half-maximal inhibitory concentration; OS, overall survival; SCLC, small cell lung cancer; ssGSEA, single-sample gene set enrichment analysis.

We next explored the relationship between the status of the CD209 signaling pathway and cisplatin sensitivity using the pRRophetic algorithm. The chemotherapeutic response prediction results showed that the predicted half-maximal inhibitory concentration (IC50) values for cisplatin were significantly lower in the George-SCLC cohort CD209-High group than in the CD209-Low group, suggesting that the activation of the CD209 signaling pathway is related to cisplatin chemosensitivity (Fig. 1C).

Our data demonstrated that the activated CD209 signaling pathway promotes the effectiveness of SCLC cisplatin chemotherapy. To further delve into the mechanisms of the contribution of the CD209 signaling pathway in SCLC, we analyzed immune-related molecule expression and immune cell infiltration between the CD209-High and CD209-Low groups (Table S4). In both the Local-SCLC and George-SCLC cohorts, CXCL9, HAVCR2, MMP9, and ICAM1 expression levels were significantly up-regulated in the CD209-High group (Fig. 1D–G; Fig. S3). This observation was consistent with that of previous studies.^4^ In addition, we evaluated the effect of the activation state of the CD209 signaling pathway on the tumor immune microenvironment through a variety of immune infiltration algorithms. The xCell score showed that M1 macrophages were up-regulated in the CD209-High group of both cohorts (Fig. 1H; Fig. S4). In the George-SCLC cohort, the absolute fraction of CD8^+^ T cells, calculated by quanTIseq, was significantly higher in the CD209-High group than in the CD209-Low group, indicating that the infiltration of CD8^+^ T cells in the activated CD209 signaling pathway was increased (Fig. 1I). The neutrophil infiltration level was further calculated using the principal component analysis (PCA) algorithm provided in the IOBR package. The neutrophil infiltration score of the CD209-High group was significantly higher than that of the CD209-Low group in both cohorts, suggesting that activation of the CD209 signaling pathway is associated with increased neutrophil infiltration (Fig. S4). Consistent with the quanTIseq results, the PCA algorithm also indicated increased infiltration of CD8^+^ T cells in the CD209-High group (Fig. S5).

Finally, we performed gene set enrichment analysis (GSEA) and ssGSEA on the expression data to further assess the effect of the CD209 signaling pathway on the regulation of various biologically relevant gene sets/signaling pathways (detailed gene sets are summarized in Table S5). The results showed that pathways related to apoptosis, drug absorption, and DNA damage repair were significantly enriched. We found that the p38MAPK cascade was significantly up-regulated in the CD209-High group of both cohorts (Fig. 1J; Fig. S6). In contrast, ABC family protein-mediated transport, DNA repair, and regulation of DNA repair were down-regulated in the CD209-High group (Fig. 1K; Fig. S7, 8). Of these, the first two pathways mentioned above were statistically significant for only the George-SCLC cohort, whereas regulation of DNA repair was significantly enriched in only the Local-SCLC cohort. We performed ssGSEA to further confirm the pathway enrichment results following GSEA. The resulting ssGSEA scores showed that multiple signaling pathways involving reactive oxygen species, negative regulation of NF-κB, inhibition of autophagy, cell cycle arrest, P38MAPK, EGFR, and negative regulation of PI3K/AKT were significantly up-regulated in the CD209-High group. These results were statistically significant in both the Local-SCLC and George-SCLC cohorts, whereas the copper ion transporter was up-regulated in only the CD209-High group of the Local-SCLC cohort (Fig. 1L; Fig. S9). This finding was consistent with previous reports^5^ and indicated that the inhibition of autophagy and DNA damage repair, coupled with the promotion of cell cycle arrest, may help improve the efficacy of cisplatin chemotherapy.

Taken together, our results suggest that the activation of the CD209 signaling pathway is associated with better OS and chemosensitivity in SCLC patients receiving cisplatin chemotherapy. Inhibition of this signaling pathway can affect the expression of immune-related molecules, alter the infiltration levels of immune cells, and regulate multiple related pathways. Possible mechanisms by which the down-regulation of the CD209 signaling pathway promotes chemoresistance may include tumor immune microenvironment, drug metabolism, apoptosis, autophagy, cell cycle arrest, and DNA damage repair. Due to the lack of adequate functional assays, more *in vitro* or *in vivo* experiments should be performed in the future to validate these findings and further investigate the pathway mechanisms.

## Conflict of interests

The authors declare that the research was conducted in the absence of any commercial or financial relationships that could be construed as a potential conflict of interests.

## Appendix A Supplementary data

Multimedia component 5 Multimedia component 5

Multimedia component 6Figure S1 Univariate and multivariate Cox regression analyses showed that activation of the CD209 signaling pathway was associated with longer OS in the George-SCLC cohort. Multimedia component 6Figure S1 Univariate and multivariate Cox regression analyses showed that activation of the CD209 signaling pathway was associated with longer OS in the George-SCLC cohort.

Multimedia component 7Figure S2 Kaplan‒Meier survival analysis for assessing the relationship between the CD209 signaling pathway activation state and OS in patients in the George-SCLC cohort. The patients were divided into two subgroups according to the median ssGSEA scores of the CD209 signaling pathway. The *P* value was calculated by the log-rank test. Multimedia component 7Figure S2 Kaplan‒Meier survival analysis for assessing the relationship between the CD209 signaling pathway activation state and OS in patients in the George-SCLC cohort. The patients were divided into two subgroups according to the median ssGSEA scores of the CD209 signaling pathway. The P value was calculated by the log-rank test.

Multimedia component 8Figure S3 Comparison of immune-related molecules between groups with different CD209 signaling pathway activation states in the George-SCLC cohort, in order of CXCL9, HAVCR2, MMP9, and ICAM1. Multimedia component 8Figure S3 Comparison of immune-related molecules between groups with different CD209 signaling pathway activation states in the George-SCLC cohort, in order of CXCL9, HAVCR2, MMP9, and ICAM1.

Multimedia component 9Figure S4 Immune cell infiltration in SCLC patients with different CD209 signaling pathway activation states. **(A)** The PCA algorithm shows the difference in the infiltration of M1 macrophage cells in the George-SCLC cohort. **(B, C)** The neutrophil infiltration score of the CD209-High group was significantly higher than that of the CD209-Low group in both cohorts. Multimedia component 9Figure S4 Immune cell infiltration in SCLC patients with different CD209 signaling pathway activation states. (A) The PCA algorithm shows the difference in the infiltration of M1 macrophage cells in the George-SCLC cohort. (B, C) The neutrophil infiltration score of the CD209-High group was significantly higher than that of the CD209-Low group in both cohorts.

Multimedia component 10Figure S5 Immune cell infiltration in SCLC patients with different CD209 signaling pathway activation states. **(A, B)** The PCA algorithm shows the difference in the infiltration of CD8 T cells in both cohorts. Multimedia component 10Figure S5 Immune cell infiltration in SCLC patients with different CD209 signaling pathway activation states. (A, B) The PCA algorithm shows the difference in the infiltration of CD8 T cells in both cohorts.

Multimedia component 11Figure S6 Pathway enrichment analyses of SCLC patients with different CD209 signaling pathway activation states. GSEA pathway enrichment results of the p38MAPK cascade in the George-SCLC cohort. Multimedia component 11Figure S6 Pathway enrichment analyses of SCLC patients with different CD209 signaling pathway activation states. GSEA pathway enrichment results of the p38MAPK cascade in the George-SCLC cohort.

Multimedia component 12Figure S7 Pathway enrichment analyses of SCLC patients with different CD209 signaling pathway activation states. GSEA pathway enrichment results for DNA repair in the George-SCLC cohort. Multimedia component 12Figure S7 Pathway enrichment analyses of SCLC patients with different CD209 signaling pathway activation states. GSEA pathway enrichment results for DNA repair in the George-SCLC cohort.

Multimedia component 13Figure S8 Pathway enrichment analyses of SCLC patients with different CD209 signaling pathway activation states. GSEA pathway enrichment results for the regulation of DNA repair in the Local-SCLC cohort. Multimedia component 13Figure S8 Pathway enrichment analyses of SCLC patients with different CD209 signaling pathway activation states. GSEA pathway enrichment results for the regulation of DNA repair in the Local-SCLC cohort.

Multimedia component 14Figure S9 ssGSEA enrichment results for multiple representative pathways from the George-SCLC cohort. The asterisks indicate differences in pathway enrichment between different CD209 signaling pathway activation states, which were detected using the Mann‒Whitney U test. ^∗∗∗∗^*P* < 0.0001, ^∗∗∗^*P* < 0.001, ^∗∗^*P* < 0.01, ^∗^*P* < 0.05. Multimedia component 14Figure S9 ssGSEA enrichment results for multiple representative pathways from the George-SCLC cohort. The asterisks indicate differences in pathway enrichment between different CD209 signaling pathway activation states, which were detected using the Mann‒Whitney U test. ∗∗∗∗P < 0.0001, ∗∗∗P < 0.001, ∗∗P < 0.01, ∗P < 0.05.

## References

[1] Kremsreiter S.M., Kroell A.S.H., Weinberger K., Boehm H. (2021). Glycan-lectin interactions in cancer and viral infections and how to disrupt them. Int J Mol Sci.

[2] Li J., Chen S., Li Y. (2022). Comprehensive profiling analysis of CD209 in malignancies reveals the therapeutic implication for tumor patients infected with SARS-CoV-2. Front Genet.

[3] George J., Lim J.S., Jang S.J. (2015). Comprehensive genomic profiles of small cell lung cancer. Nature.

[4] Wu L., Wang X., He X. (2022). MMP9 expression correlates with cisplatin resistance in small cell lung cancer patients. Front Pharmacol.

[5] Chen P., Kuang P., Wang L. (2020). Mechanisms of drugs-resistance in small cell lung cancer: DNA-related, RNA-related, apoptosis-related, drug accumulation and metabolism procedure. Transl Lung Cancer Res.

