# Supplementary material for: CD209 signaling pathway as a biomarker for cisplatin chemotherapy response in small cell lung cancer
Source: Genes Dis. 2023 Jul 16;11(3):101038. doi: 10.1016/j.gendis.2023.06.011 (PMC10806268; doi:10.1016/j.gendis.2023.06.011)
Supplement: Multimedia component 2 [file mmc2.pdf]

Supplementary Table 2. Baseline information of patients in the Local-SCLC cohort.

	CD209 DC SIGN SIGNALING-High (N=22)	CD209 DC SIGN SIGNALING-Low (N=23)	Overall (N=45)	P Value
**factor(Gender)**				1.0000
Female	4 (18.2%)	4 (17.4%)	8 (17.8%)	
Male	18 (81.8%)	19 (82.6%)	37 (82.2%)	
**factor(Smoking)**				0.7205
Non_Smoker	6 (27.3%)	4 (17.4%)	10 (22.2%)	
Smoker	16 (72.7%)	18 (78.3%)	34 (75.6%)	
Missing	0 (0%)	1 (4.3%)	1 (2.2%)	
**TMB**				0.1641
Mean (SD)	60.2 (54.1)	37.7 (34.5)	48.7 (46.0)	
Median [Min, Max]	40.2 [2.82, 192]	23.3 [4.29, 165]	30.4 [2.82, 192]	
