# Supplementary material for: CD209 signaling pathway as a biomarker for cisplatin chemotherapy response in small cell lung cancer
Source: Genes Dis. 2023 Jul 16;11(3):101038. doi: 10.1016/j.gendis.2023.06.011 (PMC10806268; doi:10.1016/j.gendis.2023.06.011)
Supplement: Multimedia component 3 [file mmc3.pdf]

Supplementary Table 3. Baseline information of patients in the George-SCLC cohort.

	CD209 DC SIGN SIGNALING-High (N=34)	CD209 DC SIGN SIGNALING-Low (N=34)	Overall (N=68)	P Value
**Age**				0.6451
Mean (SD)	64.8 (8.45)	64.2 (9.40)	64.5 (8.87)	
Median [Min, Max]	66.5 [47.0, 83.0]	63.5 [47.0, 83.0]	64.0 [47.0, 83.0]	
**Pack_Years**				0.7646
Mean (SD)	45.8 (28.8)	42.9 (21.7)	44.4 (25.5)	
Median [Min, Max]	50.0 [0, 100]	41.5 [0.750, 90.0]	45.0 [0, 100]	
Missing	9 (26.5%)	12 (35.3%)	21 (30.9%)	
