# Supplementary figures and images for: CD209 signaling pathway as a biomarker for cisplatin chemotherapy response in small cell lung cancer
Source: Genes Dis. 2023 Jul 16;11(3):101038. doi: 10.1016/j.gendis.2023.06.011 (PMC10806268; doi:10.1016/j.gendis.2023.06.011)

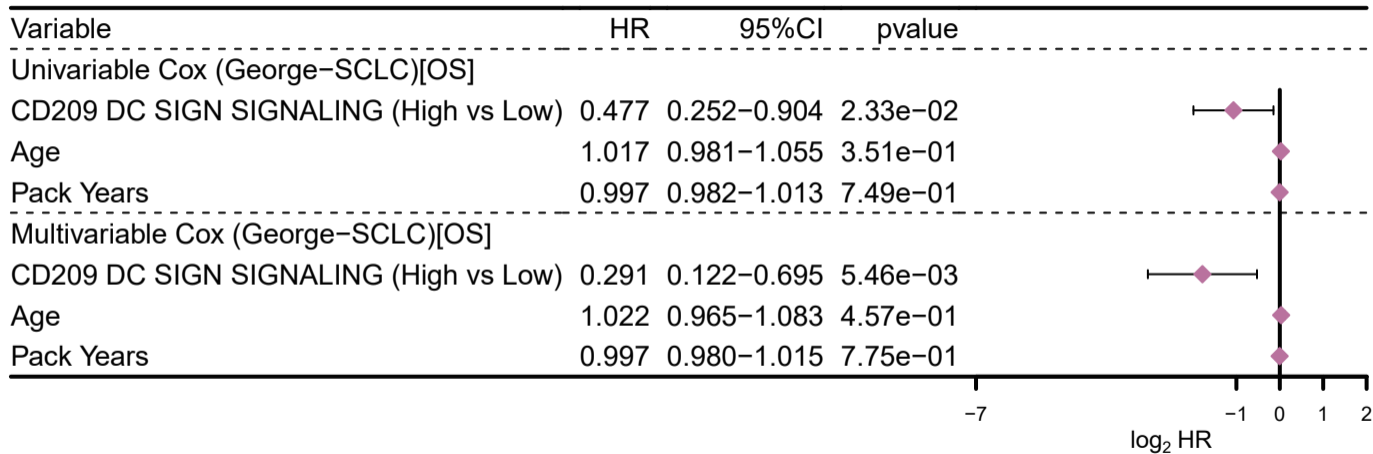

Supplement: Multimedia component 6Figure S1 Univariate and multivariate Cox regression analyses showed that activation of the CD209 signaling pathway was associated with longer OS in the George-SCLC cohort. [file mmc6.pdf]

CD209 Signaling Pathway

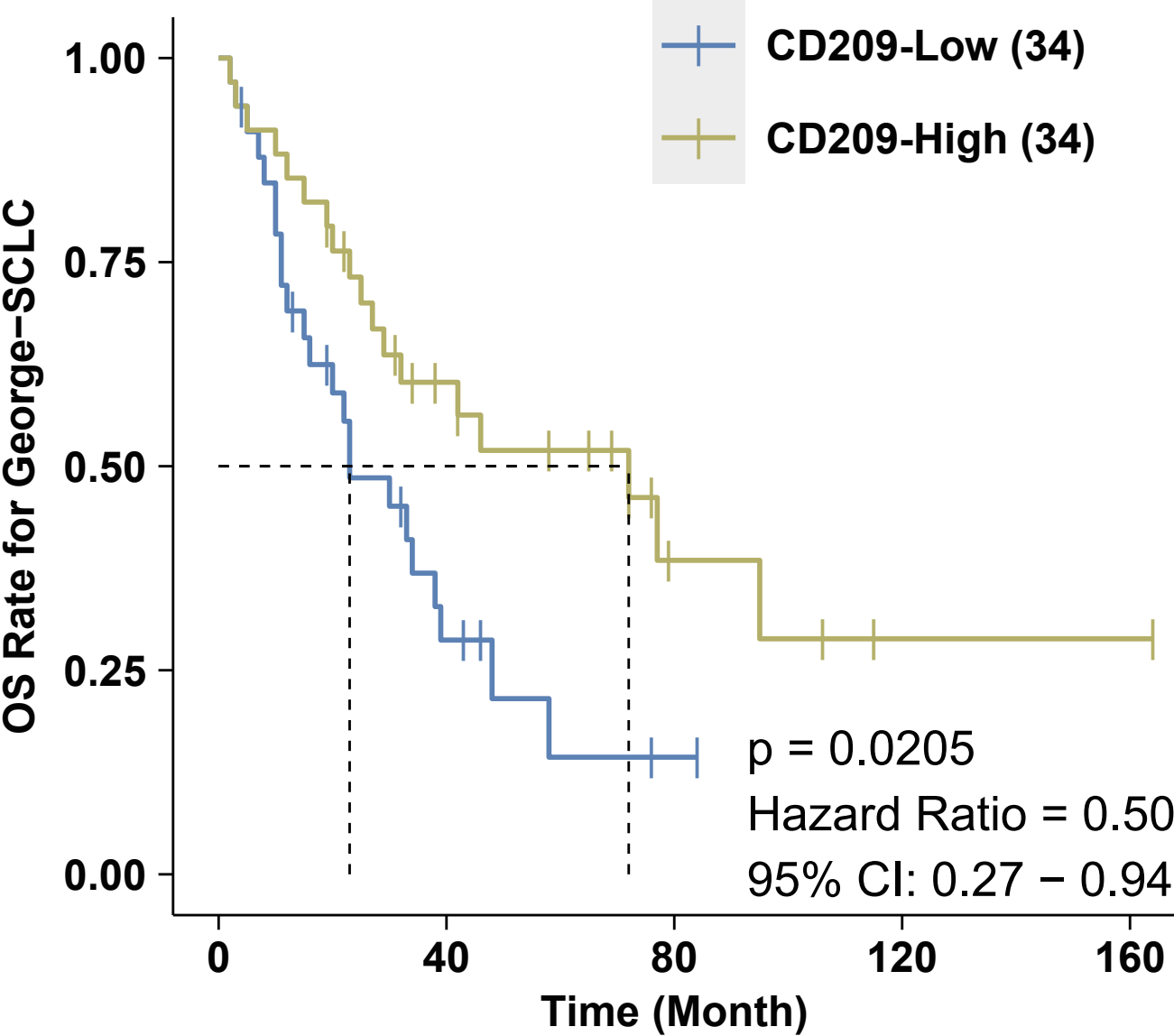

Number at risk

|    |    |   |   |   |
|----|----|---|---|---|
| 34 | 7  | 1 | 0 | 0 |
| 34 | 15 | 4 | 1 | 1 |

Supplement: Multimedia component 7Figure S2 Kaplan‒Meier survival analysis for assessing the relationship between the CD209 signaling pathway activation state and OS in patients in the George-SCLC cohort. The patients were divided into two subgroups according to the median ssGSEA scores of the CD209 signaling p [file mmc7.pdf]

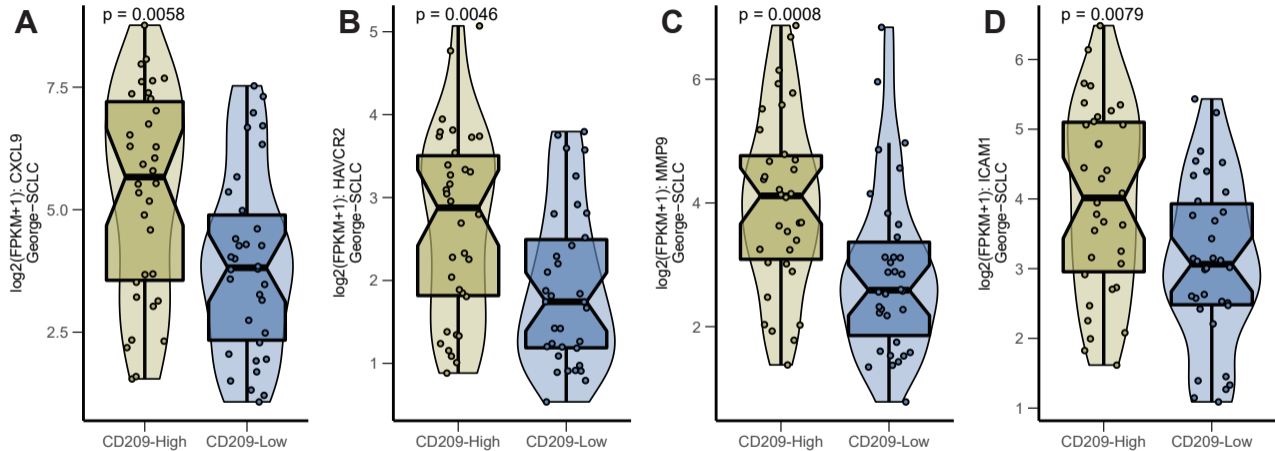

Supplement: Multimedia component 8Figure S3 Comparison of immune-related molecules between groups with different CD209 signaling pathway activation states in the George-SCLC cohort, in order of CXCL9, HAVCR2, MMP9, and ICAM1. [file mmc8.pdf]

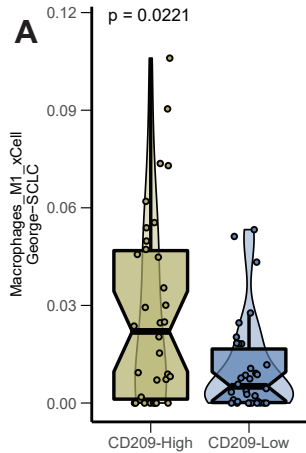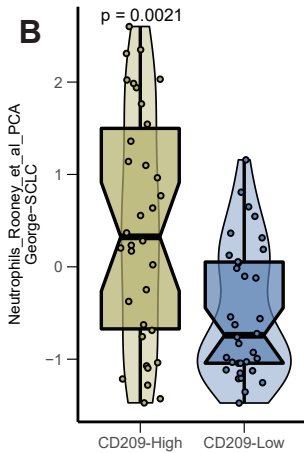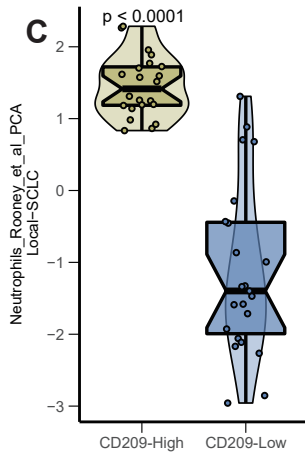

Supplement: Multimedia component 9Figure S4 Immune cell infiltration in SCLC patients with different CD209 signaling pathway activation states. (A) The PCA algorithm shows the difference in the infiltration of M1 macrophage cells in the George-SCLC cohort. (B, C) The neutrophil infiltration score of the CD209-H [file mmc9.pdf]

**(A)**CD8\_T\_cells\_Bindea\_et\_al\_PCA  
George-SCLC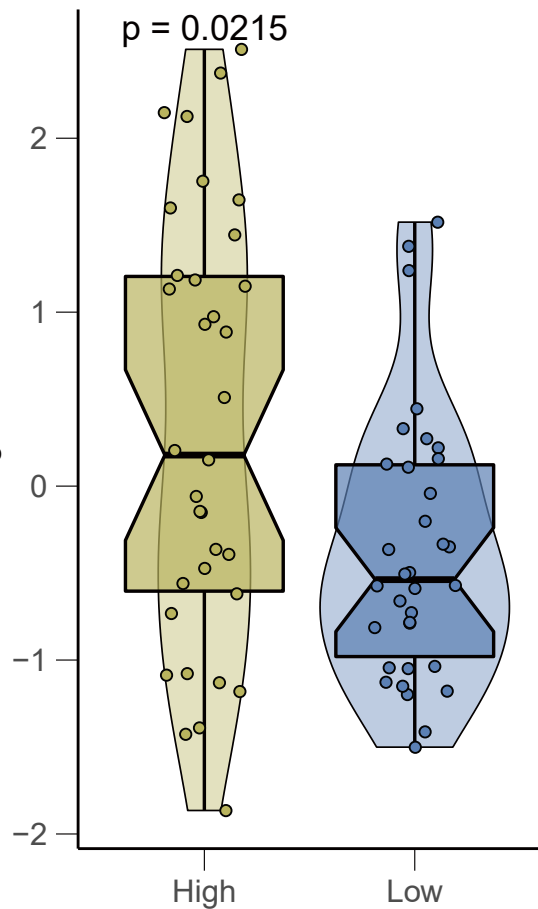**(B)**CD8\_T\_cells\_Bindea\_et\_al\_PCA  
Local-SCLC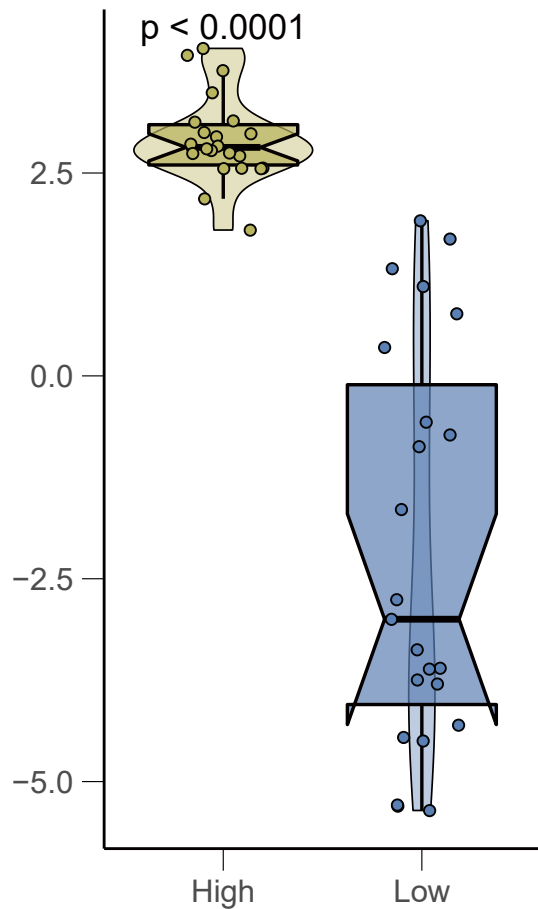

Supplement: Multimedia component 10Figure S5 Immune cell infiltration in SCLC patients with different CD209 signaling pathway activation states. (A, B) The PCA algorithm shows the difference in the infiltration of CD8 T cells in both cohorts. [file mmc10.pdf]

# CD209 DC SIGN SIGNALING

High versus Low

George-SCLC

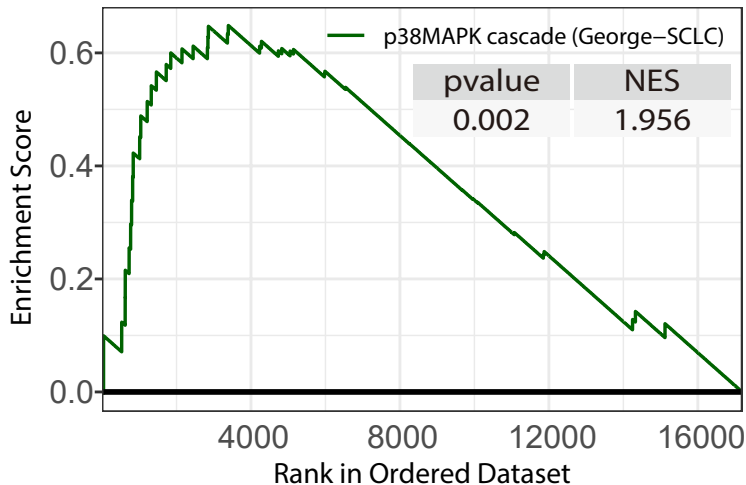

Supplement: Multimedia component 11Figure S6 Pathway enrichment analyses of SCLC patients with different CD209 signaling pathway activation states. GSEA pathway enrichment results of the p38MAPK cascade in the George-SCLC cohort. [file mmc11.pdf]

CD209 DC SIGN SIGNALING  
High versus Low  
George-SCLC

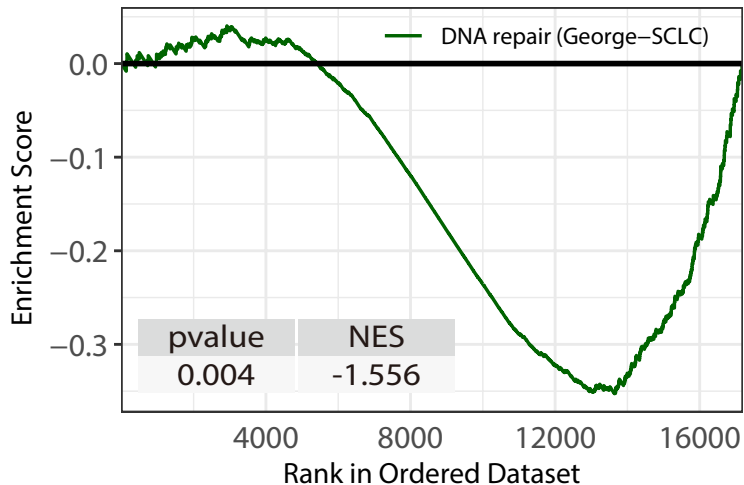

Supplement: Multimedia component 12Figure S7 Pathway enrichment analyses of SCLC patients with different CD209 signaling pathway activation states. GSEA pathway enrichment results for DNA repair in the George-SCLC cohort. [file mmc12.pdf]

# CD209 DC SIGN SIGNALING

## CD209-High versus CD209-Low

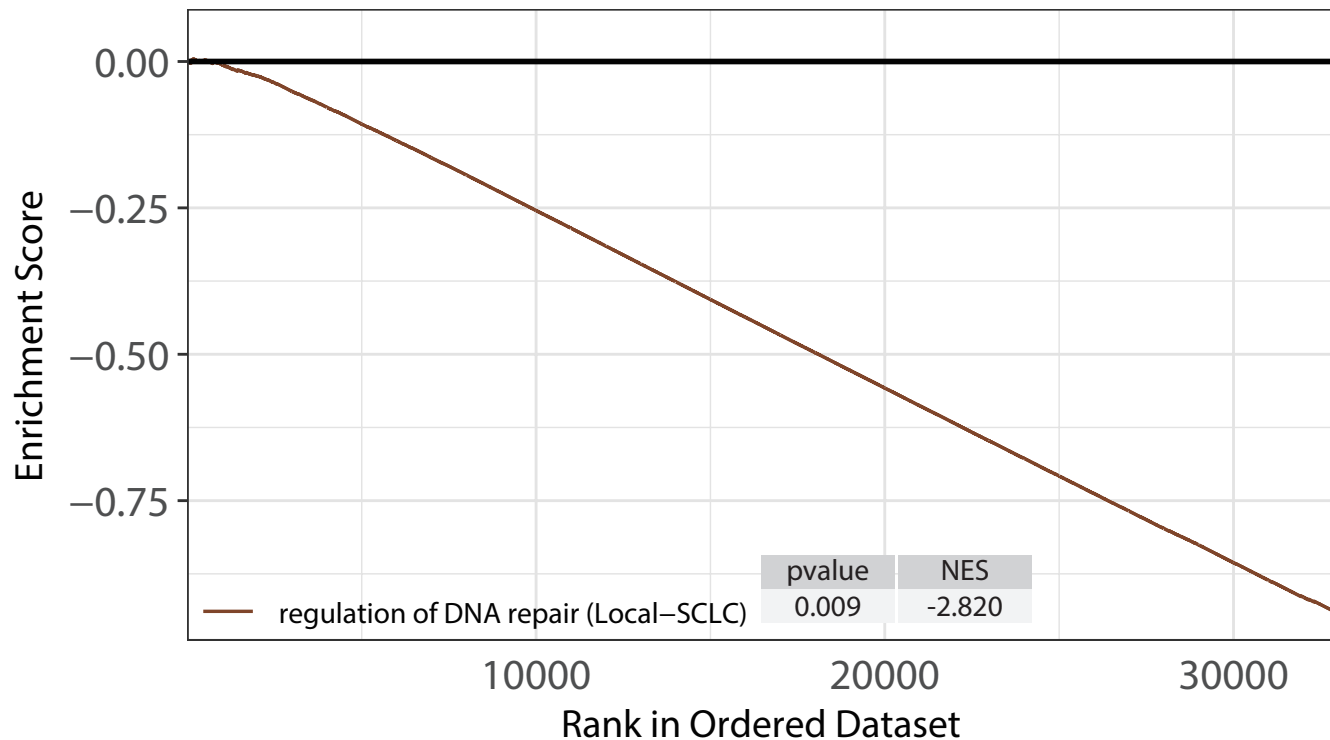

Supplement: Multimedia component 13Figure S8 Pathway enrichment analyses of SCLC patients with different CD209 signaling pathway activation states. GSEA pathway enrichment results for the regulation of DNA repair in the Local-SCLC cohort. [file mmc13.pdf]

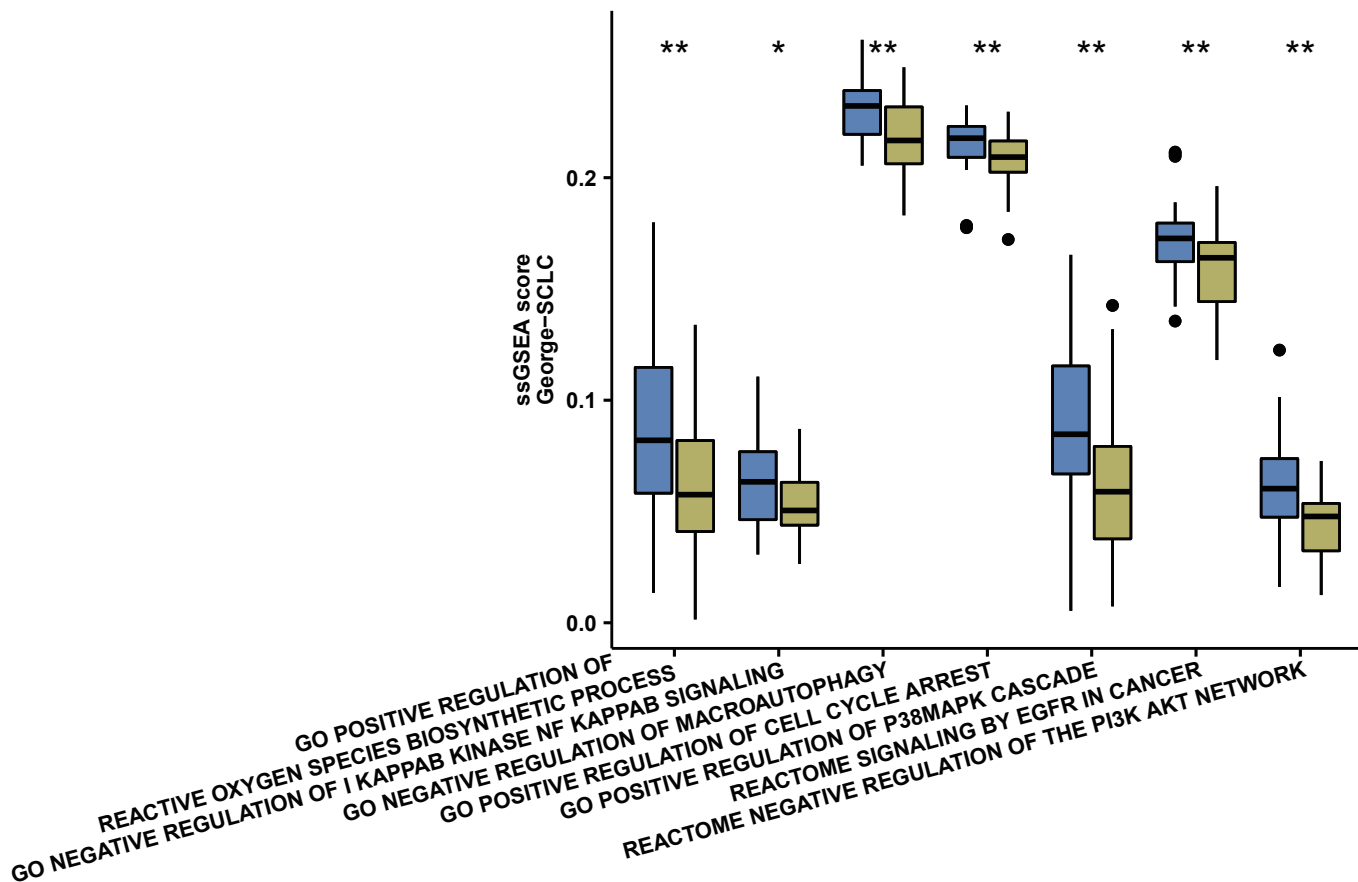

Supplement: Multimedia component 14Figure S9 ssGSEA enrichment results for multiple representative pathways from the George-SCLC cohort. The asterisks indicate differences in pathway enrichment between different CD209 signaling pathway activation states, which were detected using the Mann‒Whitney U test. ∗∗∗∗P  [file mmc14.pdf]
